# Complete Genome Sequence of Phytopathogenic *Pectobacterium atrosepticum* Bacteriophage Peat1

**DOI:** 10.1128/genomeA.00760-15

**Published:** 2015-08-13

**Authors:** Melanie Kalischuk, John Hachey, Lawrence Kawchuk

**Affiliations:** a Department of Environmental Science, Lethbridge College, Lethbridge, Alberta, Canada; b Agriculture and Agri-Food Canada, Lethbridge, Alberta, Canada

## Abstract

*Pectobacterium atrosepticum* is a common phytopathogen causing significant economic losses worldwide. To develop a biocontrol strategy for this blackleg pathogen of solanaceous plants, *P. atrosepticum* bacteriophage Peat1 was isolated and its genome completely sequenced. Interestingly, morphological and sequence analyses of the 45,633-bp genome revealed that phage Peat1 is a member of the family *Podoviridae* and most closely resembles the *Klebsiella pneumoniae* bacteriophage KP34. This is the first published complete genome sequence of a phytopathogenic *P. atrosepticum* bacteriophage, and details provide important information for the development of biocontrol by advancing our understanding of phage-phytopathogen interactions.

## GENOME ANNOUNCEMENT

The phytopathogenic Gram-negative bacterium *Pectobacterium atrosepticum* (syn. *Erwinia atrosepticum*) causes blackleg in a wide range of solanaceous plants, resulting in severe economic losses (1). Bacteriophage treatments provide an alternative strategy to control various diseases caused by bacteria. Several phages from species of *Pectobacterium* have been isolated, and a few of these have been sequenced (2–4), but a genomic sequence of a phytopathogenic *P. atrosepticum* phage has not been published. In this study, we isolated a *P. atrosepticum* phage and sequenced the entire genome of Peat1, which most closely resembles the *Klebsiella pneumoniae* KP34 phage (5).

Genomic DNA of *P. atrosepticum* phage Peat1 was isolated using density-gradient centrifugation with DNase I and EDTA proteinase K extractions (6). The genomic DNA was fragmented by ultrasonication, and library preparation was performed using the Illumina TruSeq DNA sample preparation kit. Paired-end sequencing was performed on an Illumina HiSeq 2000. Sequences were filtered for low-quality readings using the Dynamic Trim Perl script within SolexaQA. Short reads were assembled using SOAP*denovo* (http://soap.genomics.org.cn). The high-throughput platform produced 50 Mb of sequence, providing >500-fold coverage (CD Genomics, Shirley, NY). Prediction of open reading frames (ORFs) and their confirmation were obtained with Glimmer version 3.02 (7) and the Conserved Domain Database (8). Analyses of conserved protein domains were performed using BLASTp (9), and tRNAs were predicted with the use of the tRNAscan-SE software (10).

The complete double-stranded DNA genome of *P. atrosepticum* phage Peat1 consists of 45,633 bp, with a G+C content of 48.9%, 61 predicted open reading frames (ORFs), and no tRNAs. The genomic sequences of Peat1 showed little similarity to those of previously reported *Pectobacterium* phages. Consequently, 29 of the 61 ORFs encode hypothetical proteins, and the others appear to encode proteins with conserved domains or similarity to intracellular trafficking and secretion proteins, DNA/RNA polymerases, phage-related lysozyme, a DNA-binding domain in transcriptional regulators, an endo- and exonuclease, acetylornithine deacetylase, subtilisin-like serine proteases, Zn peptidases, a tail spike protein, a phage capsid domain, and phage-related proteins whose functions remain unknown.

### Nucleotide sequence accession number.

The entire genome sequence of *P. atrosepticum* phage Peat1 has been deposited in GenBank under the accession no. KR604693.
